# Supplementary material for: COZOID: contact zone identifier for visual analysis of protein-protein interactions
Source: BMC Bioinformatics. 2018 Apr 6;19:125. doi: 10.1186/s12859-018-2113-6 (PMC5889581; doi:10.1186/s12859-018-2113-6)
Supplement: Supplementary file 2 — Software build. Executable binary file of the software tool. (ZIP 118,784 kb) [file 12859_2018_2113_MOESM2_ESM.zip › cozoid/platform/docs/testng-6.5.1-javadoc.zip!/org/testng/annotations/Parameters.html]

Parameters (TestNG)


---


|  |  |  |
| --- | --- | --- |
| **Overview** \| **Package** \| **Class** \| **Tree** \| **Deprecated** \| **Index** \| **Help** | |  |
| **PREV CLASS**   **NEXT CLASS** | **FRAMES**    **NO FRAMES**     **All Classes** |  |
| SUMMARY: REQUIRED \| OPTIONAL | DETAIL: ELEMENT |  |


---


## org.testng.annotations Annotation Type Parameters

---

``` @Retention(value=RUNTIME) @Target(value={METHOD,CONSTRUCTOR,TYPE}) public @interface Parameters ```

Describes how to pass parameters to a @Test method.

**Author:**
:   Cedric Beust

---

| **Optional Element Summary** | |
| --- | --- |
| `java.lang.String[]` | `value`             The list of variables used to fill the parameters of this method. |

### value

```
public abstract java.lang.String[] value
```

:   The list of variables used to fill the parameters of this method.
    These variables must be defined in your testng.xml file.
    For example

    `@Parameters({ "xmlPath" })  
    @Test  
    public void verifyXmlFile(String path) { ... }` 

    and in testng.xml:

    `<parameter name="xmlPath" value="account.xml" />` 

    :   **Default:**: {}


---


|  |  |  |
| --- | --- | --- |
| **Overview** \| **Package** \| **Class** \| **Tree** \| **Deprecated** \| **Index** \| **Help** | |  |
| **PREV CLASS**   **NEXT CLASS** | **FRAMES**    **NO FRAMES**     **All Classes** |  |
| SUMMARY: REQUIRED \| OPTIONAL | DETAIL: ELEMENT |  |


---
